# Reconciling ACEA and MCDA: is there a way forward for measuring cost-effectiveness in the U.S. healthcare setting?

**DOI:** 10.1186/s12962-021-00266-8

**Published:** 2021-03-01

**Authors:** Bernarda Zamora, Louis P. Garrison, Aig Unuigbe, Adrian Towse

**Affiliations:** 1 Office of Health Economics, Southside, 105 Victoria Street, London, SW1E 6QT UK; 2 The Comparative Health Outcomes, Policy, and Economics (CHOICE) Institute, University of Washington, Magnuson Health Sciences Building, H Wing, H-375, Box 357630, 98195 Seattle, WA USA

**Keywords:** QALY, Augmented Cost-Effectiveness Analysis, Multi-criteria Decision Analysis, Value trade-off

## Abstract

**Background:**

The ISPOR Special Task Force (STF) on US Value Assessment Frameworks was agnostic about exactly how to implement the quality-adjusted life year (QALY) as a key element in an overall cost-effectiveness evaluation. But the STF recommended using the cost-per-QALY gained as a starting point in deliberations about including a new technology in a health plan benefit. The STF offered two major alternative approaches—augmented cost-effectiveness analysis (ACEA) and multi-criteria decision analysis (MCDA)—while emphasizing the need to apply either a willingness-to-pay (WTP) or opportunity cost threshold rule to operationalize the inclusion decision.

**Methods:**

The MCDA model uses the multi-attribute utility function. The ACEA model is based on the expected utility theory. In both ACEA and MCDA models, value trade-offs are derived in a hierarchical model with two high-level objectives which measure overall health gain separately from financial attributes affecting consumption.

**Results:**

Even though value trade-offs can be elicited or revealed without considering budget constraints, we demonstrate that they can be used similarly to WTP-based cost-effectiveness thresholds for resource allocation decisions. The consideration of how costs of medical technology, income, and severity of disease affect value trade-offs demonstrates, however, that reconciling decisions in ACEA and MCDA requires that health and consumption are either complements or independent attributes.

**Conclusions:**

We conclude that value trade-offs derived either from ACEA or MCDA move similarly with changes in main factors considered by enrollees and decision makers—costs of the medical technology, income, and severity of disease. Consequently, this complementarity between health and consumption is a necessary condition for reconciling ACEA and MCDA. Moreover, their similarity would be further enhanced if the QALY is used as the key attribute or anchor in the MCDA value function: the choice between the two is a pragmatic question that is still open.

## Background

Over the last 5 years, a number of organizations have developed new “value frameworks” proposed to support health technology assessment (HTA) in the U.S. Given some confusion about these in the HTA user community, an ISPOR Special Task Force (STF) reviewed five frameworks to try to understand and explain their similarities and differences [1]. First and importantly, the STF pointed out that they differ in the questions they are addressing—labelled as “decision context” [2]. For example, some aim to support the patient-physician interaction for clinical shared decision-making, others focus on developing clinical guidelines, and others on the issue of formulary and health benefit inclusion. In terms of methods, some recent frameworks build on cost-effectiveness analysis (CEA), some employ multi-criteria decision analysis (MCDA), and others use a multidimensional qualitative ranking. As a group of health economists, the STF focused on the issue of including a new technology in the health plan benefit, and recommended that U.S. health plans should utilize the health gain measure of the quality-adjusted life year (QALY) as the denominator in the overall cost-effectiveness metric of the cost-per-QALY gained as a starting point in their deliberations. The STF was agnostic about exactly how to implement this metric, and offered two major alternative approaches—augmented cost-effectiveness analysis (ACEA) and MCDA—while emphasizing the need to apply either a willingness-to-pay (WTP) or opportunity cost threshold rule to operationalize the inclusion decision.

In this paper, we explore whether resource allocation decisions based on either ACEA or MCDA render similar results to each other from the application of a threshold rule based on value trade-offs between financial losses and health gains generated by providing insurance coverage for a new medical technology. The initial concept of trade-off is the incremental cost-effectiveness ratio (ICER), as defined in CEA, that measures the costs incurred by the health system to gain a QALY from the treatment with a new medical technology. The CEA rule results in funding a new medical technology if its ICER is lower than a socially optimal WTP threshold which is the trade-off representing the marginal value of health, i.e., the amount that someone, the health system, or society, is willing to pay or give up in resources to obtain one more unit of health gain. Therefore, the decision maker is indifferent between reimbursing or not reimbursing a medical technology whose ICER equals the WTP threshold.

Given that WTP is a value trade-off between attributes of two different alternatives, the concept can also be used in MCDA. The “indifference condition” between having or not having access to the medical technology implies, in an MCDA context, that the decision maker is willing to pay the maximum for the health gains and health related attributes derived from the new medical technology. We therefore use the term value trade-off to represent both the WTP in ACEA and the standard value trade-off in MCDA. We value trade-offs in the ACEA model context using expected utility theory (EUT) and in the MCDA model using multi-attribute theory (MAUT) from [3].

This paper aims to reconcile decision-making on the basis of either ACEA or MCDA value trade-offs: will they yield different results, and if so, when? We analyse how the value trade-offs change with changes in income, costs of the medical technology, severity of disease, and risk attitudes. Given that ACEA and MCDA value trade-offs are affected similarly by changes in income, costs of the medical technology, and severity of disease, we consider whether changes in decision-making formulary due to budget changes and/or considering severity would be identically informed by either ACEA or MCDA metrics.

### Formulary decision-making and the QALY

This analysis concerns the decision of a health plan whether or not to make an innovative new medicine available to plan members through its drug formulary. Like the STF, we see the QALY as the core metric to support negotiations on formulary inclusion and price (since they are related) for new medicines. Well-functioning healthcare systems provide health insurance (i.e. financial protection) for the costs of treatable, financially-catastrophic illnesses. As a consequence there is no straightforward way to use market prices to assess value for patients since purchases are made through the veil of insurance.

The QALY enables us to think of the impact of new medicines or healthcare interventions in two dimensions—length of life and health-related quality of life (called “utility”). The QALY index combines the survival curve with the impact on daily quality of life and then cumulates these over time. In application, this usually entails assumptions—not all of which may be valid—particularly that there is a constant trade-off between these two over time: for example, an elderly person who is near death may care more about quality than quantity of life as compared to a younger person. Despite its limitations, both the STF and U.S. Second Panel on Cost-Effectiveness [4] called for use of the QALY as the foundation for formulary decision-making to build upon.

Furthermore, the STF emphasized the need to apply either a WTP or opportunity cost threshold, depending on the decision context of health benefit inclusion. It also recommended two alternative approaches to build on the QALY: ACEA and MCDA. The ACEA approach was reflected in ten “novel elements of value” [5, 6]. The underlying assumption was that ACEA should attempt to approach a kind of CBA—called “net monetary benefit” (NMB)—by monetizing these novel elements in some way that uses a rate of exchange between the monetary and non-monetary elements that is relevant for the decision maker. The currency of that exchange could be either QALYs or money. On the other hand, the STF also recommended experimenting with MCDA since it may do a better job of placing a relative value on broader societal impact attributes such as equity. Finally, the STF recognised that both approaches are not precise algorithms to yield the correct answer but rather tools to support the deliberative process that usually occurs in healthcare systems making these decisions.

Use of the QALY as a basis for CEA has been justified from the perspective of both major variants of microeconomic approach to health economics: welfare economics and extra-welfarism. We focus in this paper on the welfarist approach where the individual or the decision maker is maximizing his or her utility. It has long been demonstrated that under certain assumptions [7], in theory, individuals could use a decision rule that compares the cost-per-QALY impact of an intervention to their WTP threshold when choosing a health intervention. Given the existence of health insurance, people will in practice be using their consumption versus health trade-off to choose their health insurance policy, a result that authors characterize as extending their results to the purchase of an actuarially fair insurance policy: i.e., this willingness to pay affects the choice of insurance policy that a citizen makes. The resulting decision rule compares the incremental cost-effectiveness ratio (ICER) to the WTP threshold—assumed to be optimal for plan members given their insurance plan’s budget constraint.

### Defining CEA, ACEA, and MCDA

Both CEA and MCDA have been applied to a wide range of resource allocation decisions for many decades, and for our purposes, it is important to note that neither requires the use of the QALY. However, the most commonly used form of CEA in healthcare considers incremental outcomes in terms of QALYs gained and expresses costs per QALY as the ratio between incremental costs over incremental QALYs. MCDA could be seen as a broader method for weighing a wider range of multiple criteria that are relevant to a particular choice decision. The criteria need not be directly commensurable, but can be combined by assigning them relative weights and then evaluating specific alternatives as a weighted value function depending on how they vary on each of the included criteria. Indeed, since CEA involves multiple criteria—outcomes and costs—it could be thought of as a type of MCDA. In practice, applications of MCDA usually involve a larger set of criteria.

For our purposes, the key feature in both models is the possibility of defining a value trade-off between attributes. This is usually expressed as a threshold ratio between costs and QALYs in the CEA model and serves as a resource allocation decision rule. MCDA commonly uses value trade-offs to define a prioritisation decision rule according to a weighted value function. It has been argued that one of the advantages of MCDA over CEA is the transparency of trade-offs between attributes as derived from elicited weights given by decision makers, allowing explicit integration of objective measurement and value judgement [8–10]. In contrast, trade-offs measured in CEA are focused on comparing costs and QALY gains while the trade-off with other attributes and stakeholder views are implicit or not defined. Yet, transparency of MCDA requires an elicitation mechanism and group decisions, while CEA is first an analytic tool producing results that decision makers can consider.

#### ACEA—Novel elements of value

**Fig. 1:**
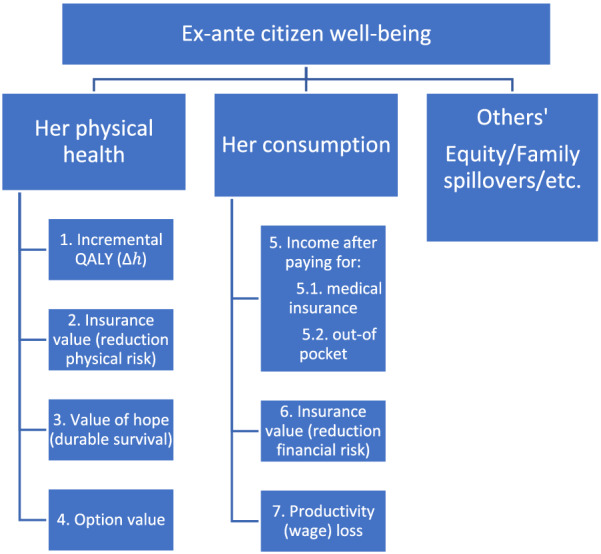
Hierarchical structure of novel elements of value

ACEA has been proposed as a method to extend CEA to including novel elements of value such as insurance value, option value, and the value of hope (see summary of studies in [11]). The inclusion of new attributes requires consideration of the trade-offs among them. As noted above, the application of ACEA monetizes all elements of value into NMB applying a common exchange rate of cost-per-QALY to all health gains (previously converted into equivalent QALYs). Health gains obtained from other attributes, such as option value [12], equity [13], insurance value derived from reducing physical risk uncertainty [14], have been measured in equivalent QALYs, or risk-adjusted QALYs. The aggregation of all health attributes into a QALY measure assumes that the individual utility function is separable between health and non-heath attributes. In this sense, the novel elements of value can be organized hierarchically as in Fig. 1.

This hierarchical framework in effect extends the CEA value trade-off between costs and QALYs to ACEA by considering two possible numeraires—one that converts all types of health gain into adjusted QALYs, and the other that converts financial attributes into consumption changes.

The translation of this hierarchical structure into the ACEA relates to the concept of separability and requires that all attributes are partitioned in two separable groups: the vector of *m* health attributes, and consumption *c*. Therefore, preferences are separable and there exists a real-value function *f* that aggregates the health attributes, e.g., in QALYs or adjusted QALYs. The attributes that are measured in monetary terms aggregate into consumption. The utility function can then be represented as:

$$ u\left( {c,h_{1,} h_{2,} ..,h_{m} } \right) = u\left( {c,f\left( {h_{1,} h_{2,} ..,h_{m} } \right)} \right). $$

For simplicity, we denote $f\left( {h_{1,} h_{2,} ..,h_{m} } \right) = h$.

Therefore, this hierarchical structure allows endogenous definition of the value trade-off between consumption and health, which is the purpose of our analysis. We do not need to use a single, fixed, exogenous cost-per-QALY threshold to monetize health gains—nor do we necessarily need to use NMB for ACEA.

In MCDA, hierarchical structures are used to organize multiple criteria into a lower number of high-level objectives.

#### MCDA—The theory and the practice

The strength of MCDA is its ability to handle diverse sets of critera for a specific complex decision with multiple stakeholders. Practitioners have developed group decision-making techniques, such as “decision conferencing” [15, 16], to deal with the challenges of assessing the benefit-risk balance of drugs through discussion and voting. Phelps [10] argues that MCDA helps decision convergence. Yet, a limitation is that the choice of criteria and the estimate of the relative value may depend on the stakeholders voting at the table. For formulary decisions made under a budget constraint involving comparisons of what to pay for, or whether to list, the 30 or more new medicines arriving each year, the framework needs to be robust and consistent enough for repeated use for different medicines. Traditional MCDA has often been used in one-off non-health applications to generate a numerical score that does not have intuitive meaning outside of that specific decision. For formulary decision-making, we would argue that MCDA will be more useful and capable of consistent repeated use if the scoring is anchored on the QALY gain, thus producing an intuitive metric of QALY-equivalents gained, e.g., in terms of healthy months or healthy years of life gained.

In practice, MCDA for healthcare decision has mostly been about prioritisation rules for broad disease-specific strategies—used to rank alternatives—rather than for technology-specific resource allocation. A recent literature review on MCDA models which consider the trade-offs between costs and benefits of medical technologies [17] found that the most frequently used criteria were health outcomes, disease impact, and implementability of the interventions. Including economic aspects was more variable, with some studies directly comparing costs against the above clinical attributes, while other studies included economic considerations in a second step.

When measuring benefits by health outcomes, 12 different attributes were considered in the different studies reviewed in [17], with the QALY not being directly considered as the unique health outcome. However, some studies have considered health outcomes (if not the QALY)—for example:

- in the EVIDEM MCDA framework [18].
- in a “performance matrix” [19, 20], or.
- in the Advance Value Framework [8].

All of these MCDA frameworks include some criteria that are elements of the conventional QALY such as therapeutic outcomes on efficacy and effectiveness, safety and tolerability, and patient-reported outcomes.

Attributes measuring costs are also sometimes included in healthcare MCDA models. In a hypothetical case study, Thokala and Duenas [21] propose an MCDA model that aggregates five different elements of value including cost-effectiveness, although Thokala et al. [19] note that including cost-effectiveness as an attribute in MCDA is contentious—and, we would argue, inadvisable. The other four key elements or criteria are: equity, innovation, patient compliance, and quality of evidence. Similarly, Diaby and Goeree [22] present different MCDA methods for four attributes used by the Canadian HTA for the Province of Ontario: costs, feasibility of adoption, consistency with expected societal and ethical values, and clinical impact. The QALY was not included.

Although the MCDA models cited above are only used to rank alternatives, Philips and Bana et Costa [23] use a similar prioritisation model which balances benefits and costs for resource allocation, including MCDA as one possible normative decision model. Also, Phelps and Madavan [24] expand the concept of cost-per-QALY threshold to account for non-QALY attributes to guide resource allocation using an MCDA framework. These approaches are compararable to the cost-per-QALY metrics used in CEA and ACEA. Moreover, they separate health attributes from non-health attributes which can be considered as a hierarchical decision model with two final major categories of QALYs and consumption, as depicted in Fig. 1. In MCDA, this hierarchical separability is related to the notion of risk independence, also known as utility independence, between two attributes of a multi-attribute utility function [25]. This is equivalent to the condition explained above for ACEA: the existence of a real function that aggregates health attributes.

Importantly, elicitation of MCDA weights and the concept of value trade-offs are based on the indifference condition: “Making judgments about how much you would give up of one objective to achieve specific amounts of other objectives is the essence of value trade-offs.” [26]. MCDA models do not directly apply any maximization or optimality condition: the underlying condition is this indifference.

#### Contributions to more consistent formulary decision-making

To move the research and policy agenda forward, we argue that it would be helpful to compare ACEA with an MCDA that includes the QALY as the anchor or key attribute. This may seem obvious to many, but in practice, other MCDA (sans QALY) approaches are up and running and are common. And at this point, it is an open question as to which of the additional (novel) elements are most important to include. We have not seen a QALY-anchored MCDA applied to new medicines. Bizarrely, there is even some debate as to whether to include the cost-effectiveness ratio or even cost as a criterion in MCDA, with others sharing our view that this should be dismissed [19, 27]. In the case of new medicines, price is endogenous, so including it would defeat the purpose of determining value. In applying MCDA to new patent-protected medicines, it makes more sense to compare the value function to a value trade-off equivalent to a value threshold.

## Methods

### Value Trade-Offs in MCDA

MCDA seeks to construct an objective function that captures all possible value trade-offs between the attributes that represent the objectives of the decision maker. These value trade-offs can be derived if the decision maker specifies two alternatives between which they are indifferent. The corresponding value trade-off between two attributes is measured as the decrease in one attribute that is compensated by the increase in other attribute. Keeney [26] explains that value trade-offs are elicited by uniquely considering preferences and objectives of the decision maker.

An important first consideration is to avoid mistakes in the definition of trade-offs in MCDA [26]. Value trade-offs measure trade-offs among attributes along the indifference curve for any level of utility. These can be elicited explicitly when two indifferent alternatives are chosen, or implicitly, after constructing the objective function from elicited weights. For example, these “swing” weights rank the importance of the ranges of the attributes, but weights do not measure value trade-offs. Rather, value trade-offs can be derived from an objective function represented by utility and fixing the utility level. This is the approach we use below to obtain value trade-offs from the multi-attribute utility function (MAUT).

### The multi-attribute utility function

A consumer’s preferences may involve a preference for achieving the maximum for one (predominant) attribute. Achieving a satisfactory level in this attribute makes achieving a satisfactory level in the second attribute less important. Let us assume that the predominant attribute is health gain, measured using QALYs. Let us also assume that we want to construct the objective function of a decision maker who elicits utilities and weights to rank the two attributes. To do this, the typical trade-off task to determine swing weights in MCDA uses probabilistic scaling (indifference probability), which seeks indifference between the choice of a certain alternative and a lottery between the two attributes.

The objective function, expressed as MAUT, considers two attributes measuring final objectives: consumption and health *(c,h).* To derive indifference probabilities, the range of these attributes (minimum and maximum levels) must be pre-determined. In our case, health is measured as QALY gains from the sick state, with a minimum of zero and maximum corresponding to cure, that is: (h^0^ = 0, h^*^ = h^w^– h^s^), with *w* for well and *s* for sick states. For consumption, the minimum level is also assumed at zero, and the maximum as maximum income in the well state: (c^0^ = 0, c^*^ = y^w^*).* Also, it is assumed that the decision maker has a monotonically increasing single-attribute utility function for each attribute: u^c^(c), u^h^(h), with u^c^(0) = u^h^(0) = 0 and u^c^(c^*^) = u^h^(h^*^) = 1.

If consumption and health are mutually utility independent—as they are if consumer welfare follows the hierarchical structure depicted in Fig. 1—the multi-attribute utility function can be represented by the multiplicative form.

$$ V\left( {c,h} \right) = k_{c} u^{c} \left( c \right) + k_{h} u^{h} \left( h \right) + Kk_{c} k_{h} u^{c} \left( c \right)u^{h} \left( h \right) $$

$k_{c}$ and $k_{h}$ are scaling constants which satisfy $0 \le k_{j} \le 1, j = c,h$, and *K* is an additional scaling constant which captures interaction between consumption and health, satisfying

$$ K = \frac{{1 - k_{c} - k_{h} }}{{k_{c} k_{h} }} .$$

The weights, known as scaling constants in MAUT, are calculated in the following way [3] (Keeney and Raiffa 1976). $k_{h}$ is derived by eliciting a subjective probability from the decision maker such that the certain alternative (c^0^, h^*^) = (0, h^w^– h^s^) is indifferent to a lottery which assigns the best (c^*^,h^*^) with probability $k_{h}$, and the worst (c^0^, h^0^) with probability $1 - k_{h}$. The scaling constant for consumption is similarly elicited as an indifference probability by comparing the lottery with the certain alternative (c^*^, h^0^) = (y^w^, 0). Consequently, $k_{c}$ and $k_{h}$ each represent the relative weight of a change in one attribute from its worst to its best level on overall utility. If health is the predominant attribute, $k_{h} > k_{c}$. Of note, the scaling constants depend on the range of each attribute because the indifference probabilities or relative weights assigned to a change from the worst to the best level are larger the larger the attribute range. For example, the scaling constant for health is expected to be larger for more severe diseases with a larger value for h^*^ = h^w^– h^s^.

Then, the resulting multi-attribute utility function can be used to measure the value trade-offs by fixing the utility level for example, as the one corresponding to the indifference curve of the status quo, when there is no access to the medical treatment. By differentiating the utility function $V\left( {c,h} \right)$ and equating the differential to zero since the utility is constant, we calculate the value trade-off as follows as the slope of any indifference curve $V\left( {c,h} \right)\text{ = }\overline{v} ,$ where $\frac{\partial V}{\partial c}dc + \frac{\partial V}{\partial h}dh = 0{:}$

$$\frac{dc}{dh} =\frac{{\partial V\text{/}\partial h}}{{\partial V\text{/}\partial c}}= \frac{{u^{{h{\prime }}} }}{{u^{{c{\prime }}} }}\frac{{\left[ {k_{h} + \left( {1- k_{c} - k_{h} } \right)u^{c} \left( c \right)} \right]}}{{\left[ {k_{c} + \left( {1 - k_{c} - k_{h} } \right)u^{h} \left( h \right)} \right]}}\left[ {MAUT \, VTO} \right] .$$

The MAUT VTO is evaluated in the status quo when the treatment is not included in the health benefit package. In general, this point considers maximum consumption, with $u^{c} \left( c \right) = u^{c} \left( {y^{w} } \right) = 1,$ and a level of health corresponding to the sick state. Then, the MAUT VTO is derived considering all possible alternatives as represented by the whole ranges of health and consumption, and it is evaluated at the point of maximum consumption. In this sense, MAUT VTO represents the maximum amount of consumption that the decision maker is willing to give up for health gain. In other words, the MAUT VTO represents the optimal cost-per-QALY trade-off.

### Value trade-offs in ACEA

The definition of value trade-offs in ACEA is based on Expected Utility Theory and concept of willingness to pay. The standard definition of WTP for a medical technology only assumes indifference between having and not having the medical treatment in the sick state [28]. The maximum WTP is determined because it is bounded by the available income. We introduce the perspective of a healthy enrollee facing the prospects of future disease through expected utility [29]. Preferences are represented by the same utility function $u\left( {c^{i} ,h^{i} } \right)$ in two states of the world,$i = s$ for sick, $i = w$ for well, and that satisfy von Neumann-Morgenstern expected utility theory, so that the utility of the expected value of a lottery equals the expected utility which is linear in probabilities for the sick $\left( \pi \right)$ and well states $\left( {1 - \pi } \right)$ and is expressed as a weighted average of utilities of each state. We consider two different levels of expected utility: (1) the status quo of not having access to the medical treatment for a non-insured person (not reimbursement *NR*), and (2) the funding of the new medical treatment for an enrollee in the health insurance plan (reimbursement *R*). The corresponding $E\left( u \right)$ in each case are:

1 $$ NR: E\left( u \right) \equiv \pi u\left( {y^{s} ,h^{s} } \right) + \left( {1 - \pi } \right)u\left( {y^{w} ,h^{w} } \right) $$

2 $$ R: E\left( u \right) \equiv \pi u\left( {y^{s} - {{\varvec\Sigma }}^{s} ,h^{s} + dh^{s} } \right) + \left( {1 - \pi } \right)u\left( {y^{w} - {{\varvec\Sigma }}^{w} ,h^{w} } \right). $$

In the *NR* case, there are no medical expenses. Then, consumption is maximum in each state and equals the income levels: $c^{s} = y^{s} ; c^{w} = y^{w}$. And health remains at the initial level according to the disease state.

In the *R* case, there are medical and health insurance expenses which decrease consumption levels: $c^{s} = y^{s} - {{\varvec\Sigma }}^{s} ; c^{w} = y^{w} - {{\varvec\Sigma }}^{w}$. If the enrollee faces the disease, she receives the medical treatment and her initial health level is improved: $h^{s} + dh^{s} .$

Given the properties of $$ E\left( u \right) $$ and continuity of the two attributes, there exist levels of $$ {{\varvec\Sigma }}^{s} $$ and $$ {{\varvec\Sigma }}^{w} $$ that equate $$ E\left( u \right) $$ to the status quo *NR.* Given that consumption equals the level of income in the status quo, $$ {{\varvec\Sigma }}^{s} $$ and $$ {{\varvec\Sigma }}^{w} $$ capture the maximum amount of medical expenses the individual is willing to pay to have access to the medical technology in each state. Consequently, the marginal rate of substitution between consumption and health in the sick state, evaluated at the status quo—that is, with initial levels of consumption $$ c^{s} = y^{s} , c^{w} = y^{w} ,$$ and health level $$ h^{s}  $$—represents the maximum value trade-off of money for health or maximum WTP.

In utility theory, value trade-offs between two attributes are measured by the marginal rate of substitution. We define the value trade-off as the marginal rate of substitution between consumption and health which measures the decrease in consumption that compensates a health gain and leaves the enrollee at the same utility level as the status quo. We derive the expression of the ACEA value trade-off (ACEA VTO) in Appendix 2. The starting point is the consideration of the maximum WTP to cover all medical expenses in the case of medical insurance. Lakdawalla et al. [29] exclude medical expenses from the definition of WTP, which is denoted by $V$, and represents the additional payment over and above medical expenses, with $p$ denoting the cost of the medical technology, and $I\left( p \right)$ insurance coverage. Since the actuarially fair premium is $\pi I\left( p \right)$, the net transfer from the insurer to the sick individual is $\left( {1 - \pi } \right)I\left( p \right).$ If this amount is lower than $p$, there is a co-payment for the difference. Then, consumption for the enrollee in each state is:

$$ c^{s} = y^{s} - {{\varvec\Sigma }}^{s} = y^{s} - \left( {p - \left( {1 - \pi } \right)I\left( p \right) + V} \right) $$

$$ c^{w} = y^{w} - {{\varvec\Sigma }}^{w} = y^{w} - \left( {\pi I\left( p \right) + V} \right). $$

The ACEA VTO is obtained by making zero the differential of $E\left( u \right)$ given that the $E\left( u \right)$ level is constant at that of the status quo. The value trade-off for the enrollee is defined as money for health gain in the sick status, or how much consumption an enrollee would expect to give up for QALY gains. The ACEA VTO result is the ratio of the expected marginal utility of health for the sick over the expected marginal utility of income, in the case of complete insurance coverage. We generalize the expected marginal utility of income in [29] by introducing an adjustment factor (γ) that may be larger than one because WTP can be larger in the well than in the sick state, in general, because income is larger in the well state. Therefore, we allow for the expected marginal utility of income to equal $\pi u_{c}^{s} + \gamma \left( {1 - \pi } \right)u_{c}^{w}$ in the case of complete insurance. This is consistent with the larger value trade-off between consumption and health in the well state than in the sick state: $\frac{{dc^{w} }}{{dh^{s} }} = \gamma \frac{{dc^{s} }}{{dh^{s} }}$. Accordingly, we define the *ex ante* VTO between consumption and health as:

$$ - \frac{{dc^{s} }}{{dh^{s} }} = \frac{{\pi u_{h}^{s} }}{{\pi u_{c}^{s} + \gamma \left( {1 - \pi } \right)u_{c}^{w} }}            \left[ {ACEA \, VTO} \right]. $$

The payer is interested in the change in reimbursement as measured by the value trade-off between the costs of the medical technology and the health gain. The maximum increase in the cost of technology that the payer would expect to reimburse for QALY gains is the ratio between the marginal utility of health for the sick over the marginal utility of income (for complete insurance coverage). We define the payer’s VTO as the trade-off between the cost of the medical technology for health gain:

$$ \frac{dp}{{dh^{s} }} = \frac{{u_{h}^{s} }}{{\pi u_{c}^{s} + \gamma \left( {1 - \pi } \right)u_{c}^{w} }}                \left[ {ACEA  \, PVTO} \right]. $$

The case of partial insurance coverage could be analyzed by considering $I^{\prime }\left( p \right)\text{ < }1$.

## Results

### Properties of value trade-offs in MCDA

The MCDA value trade-off (MAUT VTO) is positive since the ranges of the scaling constants, $$ k_{c} , k_{h} , $$ and of the utilities $$ u^{c} \left( c \right) $$, $$ u^{h} \left( h \right) $$ are within the unitary interval. The MAUT VTO depends on the single-attribute utility functions assessed by the decision maker, which are monotonically increasing with the level of consumption and health. Linear functions $$ u^{c} \left( c \right) $$, $$ u^{h} \left( h \right) $$ imply constant marginal utilities and represent risk neutrality. Concave functions $$ u^{c} \left( c \right), $$ $$ u^{h} \left( h \right) ,$$ with decreasing marginal utilities, represent risk aversion. The MAUT VTO also depends on the scaling constants. In particular if $$ k_{c} + k_{h} = 1, $$ utility is additive since the interaction term $$ K = 0. $$ If $$ k_{c} + k_{h} < 1, $$ then $$ K > 0; $$ conversely, if $$ k_{c} + k_{h} > 1, K < 0. $$

The MAUT VTO has the following properties, which are demonstrated in Appendix 1:

- MAUT VTO is positive.
- If the interaction term is zero or positive, MAUT VTO is non-decreasing with the level of consumption and non-increasing with the level of health.

These properties are intuitive and desirable for most decision makers. Firstly, there is a positive value trade-off which shows a willlngness to pay a monetary compensation for QALY gains. Secondly, the amount of money traded for health is larger, the higher the level of income. Thirdly, the decision maker is willing to give up more income (budget) to obtain QALY gains for those with a lower level of health, i.e., for more severe diseases.

The cases for which MAUT VTO is constant are special cases which require two properties of the MAUT objective function: (i) additive utility $\left( {k_{c} + k_{h} = 1} \right),$ and (ii) linear single-attribute utilities. This case represents a risk-neutral decision maker who decides on health independently of consumption: that is, the attributes are independent, which is an additional restriction to utility independence as assumed in multiplicative MAUT.

MAUT VTO changes with three factors that are usually considered by the decision maker and affect the enrollee’s consumption and health levels: the cost of the medical technology, income, and severity of disease.

Firstly, an increase in the cost of the medical technology may cause an increase in the insurance premium decreasing consumption and the value trade-off. This gives rise to MCDA property 1.

#### MCDA Property 1

*For a medical technology, the value trade-off between costs and health gain (QALY) decreases with the level of costs.*

In effect, an expensive technology, even if it delivers health gain at the prevailing willingness to pay, may not be preferred to add to the insurance package if the budget impact has a large (non-marginal) impact on other uses of the budget.

Secondly, income affects consumption and then the value trade-off in the same direction, assuming that the scaling constant $k_{c}$ does not change since consumption changes smoothly even with large income changes. More income leads to a greater willingness to pay for health gain. This gives rise to MCDA property 2.

#### MCDA Property 2

*For a medical technology, the value trade-off between consumption and health gain (QALY) increases with the level of income.*

Thirdly, we note that the effect of severity of disease on the value trade-off is channeled through a lower health level. By making health a more predominant attribute, which increases the scaling constant *k*_*h*_, the MAUT VTO decreases with the level of health which makes it larger for severe diseases. MAUT VTO also increases with *k*_*h*_ as demonstrated in Appendix 1. Thus, decision makers are willing to give up more income to achieve a given increase in health gain when the starting level of health is lower. This gives rise to MCDA property 3.

#### MCDA Property 3

*For a medical technology, the value trade-off between consumption and health gain (QALY) increases with the severity of disease.*

Finally, attitudes toward risk may be elicited through the scaling constants as well as through the concavity of the single-attribute utility functions. Concavity reinforces the effect of changes in MAUT VTO which makes the three MCDA properties above more pronounced for more risk-averse decision makers. An example on how attitudes towards risk affect the scaling constants is explained in Appendix 1.

The value trade-offs derived from MAUT and ACEA are similar although they are based on different methods of constructing the decision-maker’s objective function. Key to this similarity are two characteristics: (1) the decision-maker’s final objectives can be represented by two separable attributes measuring health and consumption; and (2) health and consumption are complements in the objective function.

### Properties of ACEA value trade-offs

The Appendix 2 demonstrates the following properties, which apply similarly to the sign of changes of the ACEA VTO and ACEA PVTO.

▪ ACEA VTO and ACEA PVTO are positive.

▪ If consumption and QALY are Hicksian quantity-complements, ACEA VTO and ACEA PVTO are non-decreasing with the level of consumption and non-increasing with the level of health.

According to these properties, the value trade-offs vary for different individuals according to their preferences for risk and factors that affect the levels of the consumption and health (income, costs of the medical technology, and severity of disease). We have the following three properties:

Firstly, an increase in costs of the medical technology may decrease both the ACEA VTO and the ACEA PVTO through lowering consumption by requiring a larger insurance premium or alternatively reducing the resources from an existing insurance premium available to the payer. This gives rise to ACEA Property 1.

#### ACEA Property 1

*For a medical technology, the value trade*-*off between consumption and health gain (QALY) and the value trade*-*off between costs and health gain (QALY) decrease with the level of costs.*

Secondly, since consumption increases with income, the ACEA VTO and ACEA PVTO increase with income. This gives rise to ACEA property 2.

#### ACEA Property 2

*For a medical technology, the value trade*-*off between consumption and health gain (QALY), and the value trade*-*off between costs and health gain (QALY) increase with income.*

Thirdly, the effect of severity of disease operates through a lower level of health in the sick state which causes larger ACEA VTO and ACEA PVTO since both value trade-offs decrease with the level of health. This gives rise to ACEA property 3.

#### ACEA Property 3

*For a medical technology, the value trade*-*off between consumption and health gain (QALY), and the value trade*-*off between costs and health gain (QALY) increase with the severity of disease.*

The degree of risk aversion is expressed in the ACEA model by the parameters of the utility function, with larger risk aversion resulting in larger decreases of the marginal utility function as the levels of health and consumption increase. Therefore, greater risk aversion reinforces the effects stated in the three ACEA properties above. In contrast, risk neutrality, which implies constant marginal utilities, implies constant value trade-offs for any levels of income and health.

### Comparison of value trade-off and cost-effectiveness threshold

The cost-per-QALY trade-off is the concept behind the ACEA VTO as well as of the WTP cost-effectiveness threshold which sets up the maximum cost per QALY that the society is willing to pay. The difference between the two concepts depends on the point at which the trade-off is measured. As Lakdawalla et al. [29] point out, the theoretical analysis models the marginal value of the introduction of a new technology at the point when consumption equals income. This implies that the ACEA VTO and the WTP threshold can coincide if the budget constraint is binding and the marginal utility of consumption equals the marginal utility of income.

We follow the concept of willingness to pay used in the model of cost-effectiveness of Garber and Phelps [7] which captures all the individual medical expenditures into WTP, leaving consumption as income less WTP, or maximum WTP as bounded by the income level, which is equivalent to maximize WTP subject to a binding budget constraint. Their results on the WTP cost-effectiveness threshold align with the properties of ACEA VTO. They demonstrate that the WTP threshold increases with income and with severity of disease since the marginal utility of income decreases.

Smith and Keeney [30] study the optimal value trade-off between financial losses and improvement in health in an EUT model where health and consumption are complements. In their model, the arguments of each period utility function are consumption and QALYs, which enter in a multiplicative form such that utility derived from health gains increases with consumption utility. As in our ACEA Property 2, the optimal value trade-off or marginal value of a QALY increases with income, and it also changes with preferences for risk, with more risk-averse individuals having a larger marginal value of a QALY.

### Health and consumption as final and separable attributes

Figure 1 illustrates the concept of separability or utility independence between health and non-health attributes. The traditional concept of separability of the utility function represents this hierarchical structure of the decision maker by partitioning all attributes into two separable groups: the vector of *m* health attributes, and consumption *c*. In this sense, the real function $f\left( {h_{1,} h_{2,} ..,h_{m} } \right) = h$ can aggregate all health attributes, starting with the QALY, which is a real function of two health attributes: survival and quality of life. Also, we have discussed other adjustments to the QALY that include novel elements that measure risk and uncertainty [12, 14], or equity [13]. Keeney and Raiffa [3] demonstrate that the multiplicative MAUT also represents a hierarchical structure where health and consumption are utility-independent.

### Health and consumption as complements

Separability or utility independence between health and consumption allow positive or negative interactions between consumption and QALYs, that is, *c* and *h* can be Hicksian q-complements if $u_{ch} =$ $\frac{{\partial^{2} u\left( {c,h} \right)}}{\partial c\partial h} > 0,$ and q-substitutes if $u_{ch} < 0.$ If $u\left( {c,h} \right)$ is additively separable $, u_{ch} = 0.$ However, decisions by an individual who considers health and consumption as substitutes—the larger the level of consumption, the lower the utility from health gains—can be inconsistent insofar as value trade-offs can increase or decrease with similar changes in relevant factors. Consistent resource allocation decisions require predictable changes in the value trade-offs of cost per QALY. For example, to allow inclusion of more expensive technologies for severe diseases with a large ICER, the value trade-off between costs and QALY should increase with severity of disease, that is, increase when the level of health decreases which requires consumption and QALYs to be considered independently or as complements.

## Discussion

This paper explores how to make consistent resource allocation decisions using value trade-offs between consumption and health that can be interpreted as cost-per-QALY thresholds. Consistency of decisions can be achieved by aggregating all of the attributes considered by the decision maker into either health or financial attributes. These could represent two generally accepted objectives of the welfare function (health and consumption) and facilitate the definition of value trade-offs in terms of money for health. The impact of new medical technologies on expected health changes which are considered separately by decision makers in current MCDA models, such as adverse events or severity of disease, could be aggregated, using QALYs as the overall measure of health improvement. Adjustments converted into QALYs using a relevant exchange rate can account for any relevant novel elements of value such as option value, value of hope, and physical insurance value. Other attributes that do not directly affect expected health can be translated to financial losses or gains and linked to the consumption (income) level. This separation into either health or financial attributes assumes some separability even as it allows for interaction between health and financial attributes. MCDA can be framed as a hierarchical model with these two final attributes, and the ACEA expected utility model is derived from utilities depending on these same two attributes.

The definition of the objective funtion in terms of two final attributes facilitates the derivation of value trade-offs in terms of money for health, explicitly in MCDA and implicitly in ACEA. These value trade-offs can be used as the decision criteria in formulary decision-making. Given the definition of the attributes, the value trade-offs measure cost per QALY in both the MCDA and ACEA models, and these are evaluated at the status quo which assumes no adoption of and no payment for the medical technology. Therefore, the consumption (income) that the decision maker is willing to give up for the expected health gain is maximised and the value trade-off represents the *ex ante* WTP threshold.

The health plan formulary includes medical technologies whose incremental cost-effectiveness ratio is at or below the cost-per-QALY threshold. However, the value trade-off does not result in a uniform cost-per-QALY threshold across plans since it will depend on relevant health and financial factors, as well as preferences for risk of the enrollees the plan is targeting or servicing. Our analysis of the variability of value trade-offs with income, the cost of the medical technology, and the severity of disease shows that, in theory, negative interactions between health and financial attributes can lead to inconsistent decisions, insofar as the threshold can decrease or increase after increases in income levels or severity of disease. Yet, empirically, we observe that most decision makers increase the threshold with the income level and that society is willing to pay more for an equivalent health gain for a more severe disease (which we see also in [14]) and decision makers reflect this. For this to happen requires that consumption and health are either independent or complement each other in the objective function and so the issue of inconsistency does not arise.

Although we motivated this paper from a welfarist perspective, we would argue that (1) both ACEA and MCDA can be applied in a tax-funded national health system, and (2) with the QALY as the key attribute in such an MCDA, the value trade-offs would yield similar results in practice. In a private insurance welfarist setting, a threshold based on WTP makes non-health impacts on private consumption important. In an extra-welfarist perspective, the key feature of the threshold is the concept of supply-side displacements to current health-generating interventions provided by payers that will occur to fund the new intervention of interest; the threshold represents the opportunity cost of funding new interventions. With an optimal health budget, the supply-side opportunity-cost based threshold is equal to societal WTP, as health care is funded, via government or other third-party payers, to a level that reflects citizen WTP for health gain. In this context, consumption costs can be compared with health gains in both the welfarist and the extra-welfarist settings [31]. We can find, however, that for example, in the single-payer tax-funded English NHS, the supply-side cost-per-QALY threshold used in HTA (£20,000-£30,000) is less than half the WTP for a QALY estimate of £60,000 recognised by the UK Government [32]. This suggests that the citizens’ trade off between consumption and health defining WTP may not necessarily be reflected in third party plans, particularly where these are government led. Further research is therefore needed to model trade offs in tax funded systems. Government attitude to risk may also be different to that of its citizens [33].

Although this discussion has focused on the QALY as the key criterion, it should be noted that the arguments would also apply to the disability-adjusted life-year (DALY), which has long been used by some decision-makers, particularly in the context of assessing the population-level “burden of disease”. Both the DALY and QALY are metrics that aim to combine length of life and quality of life into a unidimensional index. The metric of the cost-per-DALY averted has been used frequently in population-level resource prioritization and CEA in low- and middle-income countries [34]. Although there are some differences in estimation technique and application, they can both be used in either MCDA as a key criterion or in conventional or augmented CEA by converting the health gain by applying a CE threshold [35].

## Conclusions

We conclude that value trade-offs derived either from ACEA or MCDA move similarly with changes in main factors considered by enrollees and decision makers—the cost of the medical technology, income, and severity of disease—if health and consumption are either additively separable or complements—as Hicksian q-complements in EUT, and as a result of the positive interaction term in multiplicative MAUT. Consequently, this complementarity between health and consumption is a necessary condition for reconciling ACEA and MCDA approaches. Their similarity would be further enhanced if the QALY is used as the key attribute or anchor in the MCDA value function. The choice between the two approaches is a pragmatic question that is still open.

## Data Availability

Not applicable.

## Appendix 1: Derivation of value trade-off in MCDA model

The multiplicative MAUT assumes utility independence between health, h, and consumption. Then, there exists univariate utilities $u^{c} \left( c \right),  u^{h} \left( h \right).$ The MAUT objective function is:

$$ V\left( {c,h} \right) = k_{c} u^{c} \left( c \right) + k_{h} u^{h} \left( h \right) + Kk_{c} k_{h} u^{c} \left( c \right)u^{h} \left( h \right) $$

$k_{c}$ and $k_{h}$ are scaling constants which satisfy $0 \le k_{j} \le 1, j = c,h$, and *K* is an additional scaling constant which captures interaction between consumption, *c*, and health, *h*, satisfying

$$ K = \frac{{1 - k_{c} - k_{h} }}{{k_{c} k_{h} }} .$$

The value trade-off in terms of consumption for health can be derived as the slope of any indifference curve $V\left( {c,h} \right)\text{ = }\overline{V}$, where $\frac{\partial V}{\partial c}dc + \frac{\partial V}{\partial h}dh = 0{:}$

$$ - \frac{dc}{dh} = \frac{\partial V/\partial h}{\partial V/\partial c} = \frac{{u^{{h{\prime }}} }}{{u^{{c{\prime }}} }}\frac{{\left[ {k_{h} + \left( {1 - k_{c} - k_{h} } \right)u^{c} \left( c \right)} \right]}}{{\left[ {k_{c} + \left( {1 - k_{c} - k_{h} } \right)u^{h} \left( h \right)} \right]}}    \left[ {MAUT \, VTO} \right] .$$

Considering the MAUT properties, the range of the scalings constants, $$ k_{c} , k_{h} , $$ and the utilities, $$ u^{c} \left( c \right) $$, $$ u^{h} \left( h \right) $$ is the unitary interval. Assuming standard utlity properties, $$ u^{c} \left( c \right) $$ and $$ u^{h} \left( h \right) $$ are monotonically increasing functions $$ \left( {u^{{c{\prime }}} \text{ > 0;}u^{{h{\prime }}} \text{ > }0} \right), $$ with non-increasing marginal utility $$ \left( {u^{{c{\prime \prime }}} \le 0;u^{{h{\prime \prime }}} \le 0} \right) $$. Then, the MAUT value trade-off, MAUT VTO, has the following properties:

MAUT VTO is positive

$$ MAUT \,   VTO = \frac{{u^{{h{\prime }}} }}{{u^{{c{\prime }}} }}\frac{{\left[ {k_{h} + \left( {1 - k_{c} - k_{h} } \right)u^{c} \left( c \right)} \right]}}{{\left[ {k_{c} + \left( {1 - k_{c} - k_{h} } \right)u^{h} \left( h \right)} \right]}} = \frac{{u^{{h{\prime }}} }}{{u^{{c{\prime }}} }}\frac{{\left[ {k_{h} \left( {1 - u^{c} \left( c \right)} \right) + \left( {1 - k_{c} } \right)u^{c} \left( c \right)} \right]}}{{\left[ {k_{c} \left( {1 - u^{h} \left( h \right)} \right) + \left( {1 - k_{h} } \right)u^{h} \left( h \right)} \right]}} > 0. $$

MAUT VTO is strictly positive since both the numerator and denominator are positive.

MAUT VTO is not defined for an enrollee with maximum health $h^{*}$ and maximum consumption $c^{*}$ $\left( {u^{c} \left( {c^{*} } \right) = u^{h} \left( {h^{*} } \right) = 1} \right)$, and defined as “non-trader” or “extreme risk-averse” because she elicits $k_{h} = k_{c} = 1$ in the probabilistic scaling trade-off task. That is, the extreme risk-averse enrollee strictly prefers corner solutions, $\left( {c^{*} ,0} \right), \left( {0,h^{*} } \right)$ to the lottery, in the sense that she is only indifferent to the “lottery” which results in the best outcome $\left( {c^{*} ,h^{*} } \right)$ with certainty. In this case, both the numerator and the denominator are zero and the value trade-off cannot be elicited.

If the interaction term $\varvec{K} \ge 0,$ MAUT VTO is increasing with the level of consumption

$$ \frac{\partial }{\partial c}\left( {MAUT \, VTO} \right) = \frac{\partial }{\partial c}\frac{\left[ N \right]}{\left[ D \right]} = \frac{{u^{{c{\prime }}} u^{{h{\prime }}} \left( {1 - k_{c} - k_{h} } \right)}}{\left[ D \right]} - \frac{{u^{{c{\prime \prime }}} \left[ {k_{c} + \left( {1 - k_{c} - k_{h} } \right)u^{h} \left( h \right)} \right]\left[ N \right]}}{{\left[ {D^{2} } \right]}} \ge 0 $$

. where $\left[ N \right]$ and $\left[ D \right]$ denote the numerator and denominator of the *MAUT VTO* expression.

### *Proof*

Given that $\left[ N \right] > 0$ and $\left[ D \right] > 0$ (except that $\left[ N \right] = 0$ and $\left[ D \right] = 0$ for the extreme case defined as “non-trader” in the maximum as above).

If $$ K \ge 0 \Leftrightarrow \left( {k_{c} \text{ + }k{}_{h}} \right) \le 1, $$ $$ \frac{{u^{c'} u^{h'} \left( {1 - k_{c} - k_{h} } \right)}}{\left[ D \right]} \ge 0; $$ Since $$ u^{{c{\prime \prime }}} \le 0 \Rightarrow \frac{{u^{c''} \left[ {k_{c} + \left( {1 - k_{c} - k_{h} } \right)u^{h} \left( h \right)} \right]\left[ N \right]}}{{\left[ {D^{2} } \right]}} \le 0.$$

Therefore, $If \, K \ge 0, \frac{\partial }{\partial c}\left( {MAUT \, VTO} \right) \ge 0 .$ We can consider two cases:

Risk neutrality: $\varvec{u}^{\varvec{c}} \left( \varvec{c} \right)$ is linear, $\varvec{u}^{{\varvec{c''}}} = 0.$ In this case, $\frac{\partial }{{\partial \varvec{c}}}\left( {\varvec{MAUT}\,\varvec{VTO}} \right)$ has the same sign as the interaction term, that is, as $\left( {1 - \varvec{k}_{\varvec{c}} - \varvec{k}_{\varvec{h}} } \right).\varvec{  }$ A positive interaction term is more intuitive since it implies that WTP is larger the larger the enrollee’s consumption level. If MAUT is additive, $\left( {1 - \varvec{k}_{\varvec{c}} - \varvec{k}_{\varvec{h}} } \right) = 0,\varvec{ }$ and *MAUT VTO* does not change with the level of consumption.

Risk aversion: $\varvec{u}^{\varvec{c}} \left( \varvec{c} \right)$ is concave, $\varvec{u}^{{\varvec{c''}}} < 0.$ In this case, if the interaction term is non-negative, MAUT VTO is strictly increasing in *c*, including with additive MAUT $\left( {\frac{\partial }{{\partial \varvec{c}}}\left( {\varvec{MAUT}\,\varvec{VTO}} \right) > 0} \right).$ If the interaction term is negative, the sign of $\frac{\partial }{{\partial \varvec{c}}}\left( {\varvec{MAUT}\,\varvec{VTO}} \right)$ is ambiguous. Note that this is the standard definition of risk aversion through the utility function and it is not related to the case of a decision maker eliciting scaling constant equal to one, labelled above as “extreme risk-averse”, that results in a negative interaction term *K *= -*1*.

If the interaction term $\varvec{K} \ge 0,$ MAUT VTO is decreasing with the level of health

$$ \frac{\partial }{\partial h}\left( {MAUT  \, VTO} \right) = \frac{\partial }{\partial h}\frac{\left[ N \right]}{\left[ D \right]} = \frac{{u^{h} {\prime \prime }\left[ {k_{h} + \left( {1 - k_{c} - k_{h} } \right)u^{c} \left( c \right)} \right]}}{\left[ D \right]} - \frac{{u^{c} {\prime }u^{h} {\prime }\left( {1 - k_{c} - k_{h} } \right)}}{{\left[ {D^{2} } \right]}} $$

.

where $\left[ N \right]$ and $\left[ D \right]$ denote the numerator and denominator of the *MAUT VTO* expression.

Therefore, $If \, K \ge 0, \frac{\partial }{\partial h}\left( {MAUT \, VTO} \right) \le 0 .$ We can consider two cases:

Risk neutrality: $\varvec{u}^{\varvec{h}} \left( \varvec{h} \right)$ is linear, $\varvec{u}^{{\varvec{h''}}} = 0.$ In this case, $\frac{\partial }{{\partial \varvec{h}}}\left( {\varvec{MAUT}\, \varvec{VTO}} \right)$ has the opposite sign to the interaction term, that is, negative if $\left( {1 - \varvec{k}_{\varvec{c}} - \varvec{k}_{\varvec{h}} } \right) > 0.$ A positive interaction term is more intuitive since it implies that WTP is lower the larger the enrollee’s health level. If MAUT is additive, $\left( {1 - \varvec{k}_{\varvec{c}} - \varvec{k}_{\varvec{h}} } \right) = 0,\varvec{ }$ and MAUT VTO does not change with the level of health.

Risk aversion: $\varvec{u}^{\varvec{h}} \left( \varvec{h} \right)$ is concave, $\varvec{u}^{{\varvec{h''}}} < 0.$ In this case, if the interaction term is non-negative, MAUT VTO is strictly decreasing in *h*, including with additive MAUT $\left( {\frac{\partial }{{\partial \varvec{c}}}\left( {\varvec{MAUT}\,\varvec{VTO}} \right) > 0} \right).$ If the interaction term is negative, the sign of $\frac{\partial }{{\partial \varvec{h}}}\left( {\varvec{MAUT} \varvec{VTO}} \right)$ is ambiguous. Similarly, this concept of risk aversion operates through utility, not scaling constants.

The proofs below demonstrate the effect of increases of income (↑ *y*), costs of the medical technology (↑ *p*), and severity of disease (↓ ${\text{h}}$) on the *MAUT VTO*, considering that these effects channel through changes in consumption or health and how *MAUT VTO* changes:

*MCDA Property 1: For a medical technology, the value trade*-*off between costs and health gain (QALY) decreases with the level of costs.*

### *Proof*

*If p* ↑, c $\downarrow$ $\Rightarrow$ *MAUT VTO* $\downarrow$ (increasing in *c*).

*MCDA Property 2: For a medical technology, the value trade*-*off between consumption and health gain (QALY) increases with the level of income.*

### *Proof*

*If y* ↑, c $\uparrow$. And assume $k_{c}$ does not change because the consumption range $\left[ {0,y^{w} } \right]$ does not change $\Rightarrow$ *MAUT VTO* $\uparrow$ (increasing in *c*).

*MCDA Property 3: For a medical technology, the value trade*-*off between consumption and health gain (QALY) increases with the severity of disease.*

### *Proof*

Severity of disease can have two effects: (a) Reducing the level of health: *If h* $\downarrow \varvec{ }$ *MAUT VTO* $\uparrow$ (decreasing in *h*). (b) Increasing the range of health gain and this causes the health attribute becomes more predominant: $\varvec{k }_{\varvec{h}}$ ↑. Taking derivatives of *MAUT VTO* w.r.t $\varvec{k }_{\varvec{h}}$ ↑ gives:

$$ \frac{\partial }{{\partial k_{h} }}\left( {MAUT \, VTO} \right) = \frac{{{\text{u}}^{{{\text{h}}{\prime }}} \left[ {1 - {\text{u}}^{\text{c}} \left( {\text{c}} \right)} \right]}}{{\left[ {\text{D}} \right]}} + \frac{{{\text{u}}^{{{\text{c}}{\prime }}} {\text{u}}^{\text{h}} \left( {\text{h}} \right)\left[ {\text{N}} \right]}}{{\left[ {\text{D}} \right]^{2} }} > 0. $$

Given that $0 \le u^{c} \left( c \right) \le 1,$ the sign of the derivative is positive: *MAUT VTO* $\uparrow$.

## Appendix 2: Derivation of value trade-off in ACEA model

To demonstrate ACEA Key Properties, we analyse the changes implied for the value trade-off, which is defined as the marginal rate of substitution between consumption and health along the EU indifference curve $E\left( u \right)\text{ = }\pi u\left( {c^{s} ,h^{s} } \right)\text{ + }\left( {1\text{ - }\pi } \right)u\left( {c^{w} ,h^{w} } \right)\text{ = }\overline{EU},$ so that it is invariant to changes in health and consumption caused by access to the medical treatment, that is:

$$ \frac{\partial E\left( u \right)}{{\partial c^{s} }}dc^{s} + \frac{\partial E\left( u \right)}{{\partial h^{s} }}dh^{s} + \frac{\partial E\left( u \right)}{{\partial c^{w} }}dc^{w} = \pi u_{c}^{s} dc^{s} + \pi u_{h}^{s} dh^{s} + \left( {1 - \pi } \right)u_{c}^{w} dc^{w} = 0 $$

$$ MRS\left( {c^{s} ,h^{s} } \right) = \frac{{\partial E\left( u \right)/\partial h^{s} }}{{\partial E\left( u \right)/\partial c^{s} }} = - \frac{{dc^{s} }}{{dh^{s} }} = \frac{{u_{h}^{s} }}{{u_{c}^{s} }} + \frac{1 - \pi }{\pi }\frac{{u_{c}^{w} }}{{u_{c}^{s} }}\frac{{dc^{w} }}{{dh^{s} }} .$$

This *MRS* can be evaluated for a given value trade-off between consumption and health in the well state expressed as a proportion (γ) of the value trade-off between consumption and health in the sick state: $\frac{{dc^{w} }}{{dh^{s} }} = \gamma \frac{{dc^{s} }}{{dh^{s} }}$. Then, the value trade-off of interest is defined as as money for health gain in the sick status, or how much consumption an enrollee would expect to give up for QALY gains obtained from the medical treatment and remain indifferent to not having the treatment. ACEA *ex ante* value trade-off is:

$$ - \frac{{dc^{s} }}{{dh^{s} }} = \frac{{\pi u_{h}^{s} }}{{\pi u_{c}^{s} + \gamma \left( {1 - \pi } \right)u_{c}^{w} }}            \left[ {ACEA \, VTO} \right] .$$

Considering the medical treatment is funded through insurance coverage, consumption in the sick state is increased by the net payment of the insurance coverage which is $\left( {1 - \pi } \right)p$ with complete coverage. Therefore $c^{s} = y^{s} - \pi p$, so that medical spending decreases consumption in the sick state by:

$$ \frac{{dc^{s} }}{dp} = - \pi. $$

Therefore, the value trade-off between the cost of medical treatment and health gain defines the maximum increase in the cost of technology that the payer would expect to reimburse for QALY gains. ACEA payer’s value trade-off:

$$ \frac{dp}{{dh^{s} }} = \frac{{u_{h}^{s} }}{{\pi u_{c}^{s} + \gamma \left( {1 - \pi } \right)u_{c}^{w} }}                \left[ {ACEA \, PVTO} \right]. $$

Preferences between consumption and QALY are represented by a monotonically increasing utility function, $u\left( {c,h} \right)$, with decreasing marginal utilities: $u_{c} > 0; u_{h} > 0; u_{cc} < 0; u_{hh} < 0.$ In principle, we allow positive or negative interactions between consumption and QALYs, that is, *c* and *h* are Hicksian q-complements if $u_{ch} > 0$, and q-substitutes if $u_{ch} < 0.$ If $u\left( {c,h} \right)$ is additively separable, $u_{ch} = 0.$ Then, ACEA VTO has the following properties (the expressions for *ACEA PVTO* are similar and have the same sign).

ACEA VTO is positive. The proof is immediate since marginal utilities are strictly positive: $u_{c} > 0; u_{h} > 0.$ Then, numerator and denominator are positive. If $\varvec{u}_{{\varvec{ch}}} \ge 0$, ACEA VTO is increasing with the level of consumption:

$$ \frac{\partial }{\partial c}\left( {ACEA VTO} \right) = \frac{\partial }{\partial c}\frac{\left[ N \right]}{\left[ D \right]} = \frac{{\pi u_{ch}^{s} }}{\left[ D \right]} - \frac{{\left( {\pi u_{cc}^{s} + \gamma \left( {1 - \pi } \right)u_{cc}^{w} } \right)\left[ N \right]}}{{\left[ {D^{2} } \right]}} > 0. $$

where $\left[ N \right]$ and $\left[ D \right]$ denote the numerator and denominator of the *ACEA VTO* expression.

### *Proof*

The first term is non-negative $\left( {if \, u_{ch}^{s} \ge 0} \right).$ The second term substracts a strictly negative term. If $\varvec{u}_{{\varvec{ch}}} \ge 0,$ ACEA VTO is decreasing with the level of health:

$$ \frac{\partial }{\partial h}\left( {ACEA VTO} \right) = \frac{\partial }{\partial h}\frac{\left[ N \right]}{\left[ D \right]} = \frac{{\pi u_{hh}^{s} }}{\left[ D \right]} - \frac{{\left( {\pi u_{ch}^{s} + \gamma \left( {1 - \pi } \right)u_{ch}^{w} } \right)\left[ N \right]}}{{\left[ {D^{2} } \right]}} < 0. $$

where $\left[ N \right]$ and $\left[ D \right]$ denote the numerator and denominator of the *ACEA VTO* expression.

### *Proof*

The first term is strictly negative $\left( {u_{hh}^{s} < 0} \right).$ The second term substracts a non-negative term if $u_{ch}^{s} \ge 0,$ and $u_{ch}^{w} \ge 0.$

The proofs below demonstrate the effect of increases of income (↑ *y*), the cost of the medical technology (↑ *p*), and severity of disease (↓ ${\text{h}}$) on the *ACEA VTO*, considering that these effects channel through changes in consumption or health and analyze how *ACEA VTO* (and *ACEA PVTO*) changes:

*ACEA Property 1: For a medical technology, the value trade-off between consumption and health gain (QALY), and the value trade-off between costs and health gain (QALY) decreases with the level of costs.*

### *Proof*

Consider the consumption in the sick state for an insured individual $c^{s} = y^{s} - \pi p,  where \,\pi \, p$ is the insurance premium. This means that the net transfer to the individual in the sick state is $p - \pi p = \left( {1 - \pi } \right)p.$

*If p* ↑, $c^{s} \downarrow$ $\Rightarrow ACEA$ *VTO* $\downarrow$ and $ACEA$ *PVTO* $\downarrow$ (increasing in *c*).

*ACEA Property 2: For a medical technology, the value trade-off between consumption and health (QALY), and the value trade-off between costs and health gain (QALY) increases with income.*

### *Proof*

*If* $y^{s} \uparrow$ *and/or* $y^{w}\uparrow , c^{s}\uparrow, c^{w}\uparrow \Rightarrow ACEA$ *VTO* $\uparrow$ and $ACEA$ *PVTO* $\uparrow$ (increasing in *c*).

*ACEA Property 3: For a medical technology, the value trade-off between consumption and health gain (QALY), and the value trade-off between costs and health gain (QALY) increase with the severity of disease.*

### *Proof*

*If* $h^{s} \downarrow$ $\Rightarrow ACEA$ *VTO* $\uparrow$ and $ACEA$ *PVTO* $\uparrow$ (decreasing in *h*).

## Abbreviations

ACEA: Augmented cost-effectiveness analysis
CEA: Cost-effectiveness analysis
DALY: Disability-adjusted life year
EUT: Expected utility theory
HTA: Health technology assessment
ICER: Incremental cost-effectiveness ratio
ISPOR SFT: The Professional Society for Health Economics and Outcomes Research Special Task Force
MAUT: Multi-attribute utility theory
MCDA: Multi-criteria decision analysis
NMB: Net monetary benefit
QALY: Quality-adjusted life year
WTP: Willingness to pay
